# Longitudinal Healing and Amputation Trajectories in Diabetic Foot Ulcers: Predictive Power of Wound Area and Duration and Sample‐Size Implications From the Diabetic Foot Consortium

**DOI:** 10.1111/wrr.70189

**Published:** 2026-07-15

**Authors:** Charlotte Xu, Brian M. Schmidt, Amy Krambrink, Junyoung Park, Peter X. K. Song, Teresa L. Z. Jones, Crystal M. Holmes, Kellen Chen, Wen Ye, Giselle Kolenic, Sashwati Roy, Chandan K. Sen, Rodica Pop‐Busui, Cathie Spino, Irina Gaynanova

**Affiliations:** ^1^ School of Public Health, Department of Biostatistics University of Michigan Ann Arbor Michigan USA; ^2^ Department of Internal Medicine, Division of Metabolism, Endocrinology, and Diabetes University of Michigan Health Ann Arbor Michigan USA; ^3^ National Institute of Diabetes and Digestive and Kidney Diseases (NIDDK) Bethesda Maryland USA; ^4^ University of Arizona College of Medicine Tucson Arizona USA; ^5^ University of Pittsburgh School of Medicine Pittsburgh Pennsylvania USA; ^6^ Division of Endocrinology, Diabetes and Clinical Nutrition Oregon Health & Science University Portland Oregon USA

**Keywords:** amputation, biomarkers, clinical trials, diabetic foot ulcers, predictive modeling, wound healing

## Abstract

Diabetic foot ulcers (DFUs) are the leading cause of amputations in people with diabetes, mainly due to poor wound healing. This study evaluated DFU trial designs and analysed observational data from the two prospective Diabetic Foot Consortium (DFC) studies to estimate longitudinal healing and amputation rates, assess the effects of wound surface area and duration on healing, and inform sample size considerations for future trials. We analysed data from Open Wound Master Protocol (MP, *n* = 419) and c‐Myc Biomarker (*n* = 140). The primary outcome was complete wound healing. Time‐to‐event analysis estimated healing and amputation rates, with amputations classified as non‐healed. Logistic regression assessed the prediction of healing based on baseline wound characteristics, with model performance evaluated using AUC (area under the curve). Sample size calculations were performed to achieve 80% power. In the MP study, healing rates were 26% by week 12% and 49% by week 32; amputation rates were 4% and 10%, respectively. The c‐Myc study showed 38% healing and 2.5% amputation by week 12. Wound area and duration significantly predicted healing (AUC ≥ 0.70 by 24 weeks). For smaller treatment effects, predicted healing rates varied by up to 40% between small and large wounds, impacting sample size. Contemporary DFU trial designs and healing times remained largely unchanged over two decades. While shorter trials (e.g., 12 weeks) theoretically require smaller sample sizes due to reduced outcome variance, they risk failing to capture the full treatment effect. Trial design must account for baseline wound characteristics, which should inform eligibility criteria and follow‐up.

**Abbreviations**
ABI: ankle brachial index
AUC: area under the curve
CI: confidence intervals
DFC: Diabetic Foot Consortium
DFU: diabetic foot ulcers
EMR: electronic medical records
FDA: US Food and Drug Administration
HbA1c: Haemoglobin A1c
IPW: inverse probability weighting
IQR: interquartile range
MAR: missing‐at‐random
MP: Open Wound Master Protocol
NIDDK: National Institute of Diabetes and Digestive and Kidney Diseases
OSMB: Observational Safety and Data Monitoring
PAR: percent surface area reduction
p‐GR: phosphorylatedglucocorticoid receptor
SOC: standard‐of‐care

## 1 Introduction

Diabetic foot ulcers (DFU) remain a prevalent and serious complication of diabetes, leading to significant morbidity and mortality, high lower extremity amputation rates, and extended healthcare costs [1, 2]. Effective management and treatment of DFUs remain complex, requiring a nuanced understanding of patient factors and wound characteristics. The currently recommended standards in wound care include identification and eradication of infection, management of ischemia, appropriate wound debridement and wound dressing selection, and offloading.

Despite the wide availability of wound products used in the usual care of DFU, the last known medication approved by the US Food and Drug Administration (FDA) to treat DFU was becaplermin in 1997 [3]. Since then, many wound dressings and related wound care products have been regulated as medical devices rather than drugs. Device regulatory pathways differ from drug approval pathways; for example, the 510(k) pathway is based on substantial equivalence to a predicate device and often does not require the same type or extent of clinical efficacy evidence typically expected for drug approval, although clinical data may be required in some 510(k) submissions. Other device pathways, including premarket approval, may require more extensive evidence [4]. Thus, there remains a critical need for comprehensive clinical data to guide DFU clinical trial design, especially given the high variability of care provided and the heterogeneity in patient demographics, associated comorbidities and risk factors, and in wound characteristics among patients with DFUs. This gap has impeded the development of evidence‐based, standardised treatment protocols, placing DFU individuals at risk for poor outcomes. It has led to variation in clinical trial endpoints and outcome assessments. These issues highlight the need for evidence to guide the development of rigorous clinical trial design that accounts for diverse baseline characteristics and wound complexities.

For example, a common primary outcome measure for a DFU clinical trial assessing an intervention is healing by 12 weeks. This is an arbitrary timeline. A large DFU may respond to the trial intervention but not heal by 12 weeks. By contrast, a small DFU of short duration assigned to the control intervention may heal by 12 weeks and obscure any effect of the trial intervention. Furthermore, DFU clinical trials only seeking the 510(k) clearance often select narrow inclusion criteria enriched with lower risk DFUs to overcome the limitations of a healing outcome after a short duration. However, this leads to difficulty recruiting and poor generalizability to the broader population of patients with DFUs seen at the point of care. All these highlight the need for well‐designed clinical trials poised to objectively assess the true treatment efficacy and outcomes that are relevant across the entire clinical spectrum of DFU presentations.

Addressing these gaps in clinical trial methodology, including the need for innovative outcome measures and tailored treatment protocols, is critical for improving outcomes in DFU management and advancing the development of effective therapies.

The *primary hypothesis* for this analysis is that the current study design in DFU trials is insufficiently short and does not accurately capture complete wound healing events and rates even after adjustment for commonly‐used factors such as wound surface area and wound duration. To evaluate this hypothesis and inform trial design, we aimed to estimate healing rates over time and assess the effect of wound characteristics on healing, using prospective observational data from the ongoing Biomarkers for Active Diabetic Foot Ulcers (referred to hereafter as the Master Protocol [MP]; ClinicalTrials.gov ID NCT06104969) and a completed biomarker study (c‐Myc; NCT04591691) [5] conducted by the Diabetic Foot Consortium (DFC) [6]. Our analysis has four parts: (i) estimate healing rates at specific time intervals and compare them with existing literature [7, 8, 9, 10]; (ii) estimate time‐specific amputation rates in the Master Protocol (with amputations treated as subset of non‐healed) and contextualise them against published rates [8]; (iii) assess the effects of wound characteristics—specifically wound surface area and duration—on healing by building a predictive model for healing status and evaluating its performance; and (iv) derive sample size considerations for future trials based on expected healing rates and effect sizes.

## 2 Methods

### 2.1 Master Protocol Multisite Study

The DFC MP is a multisite observational platform study designed to examine multiple biomarkers from multiple biospecimens (e.g., tissue, plasma, serum, wound fluid urine) for their capacity to identify DFUs more likely to heal. The ultimate goal of the study is to validate biomarkers predictive of healing or nonhealing and apply findings in the clinical setting to drive personalised wound management decisions and to inform choices of wound healing interventions. The MP enrols contemporary patients with DFUs receiving standard‐of‐care (SOC) in an outpatient setting across a range of healthcare systems, including community settings and tertiary care hospitals, participating in the DFC [11]. The study is registered at ClinicalTrials.gov ID NCT06104969. The study was conducted in accordance with the Declaration of Helsinki and was approved by the Institutional Review Board of Advarra Inc. All participants signed a written informed consent permitting collection and use of data and tissue samples for the study and any ancillary analyses.

All participants are followed every other week for 1 month and monthly thereafter until the wound is healed or for 1 year (Week 52 of study) if the wound has not healed. Participants who experience wound healing before Week 52 have a confirmation visit 2 weeks later. These participants are also assessed for recurrence of their DFU at Weeks 52 and 78, respectively, via phone call or video visit. Participants who do not experience wound healing by Week 52 have an additional phone call or video visit scheduled at Week 78 to assess patient‐reported wound healing, infection, or amputation with confirmation by electronic medical records (EMR), if available. HbA1c was extracted from participants' EMR if available within 3 months of enrollment or collected at first visit if not. Comorbidities (coronary artery disease, nephropathy/CKD, retinopathy) were ascertained through chart review of participants' medical history rather than current laboratory or clinical assessment.

Eligible participants include adults 18 years or older who have a clinical diagnosis of diabetes with a foot ulcer. Exclusion criteria include participation in an interventional clinical trial for DFU within 1 month of Visit 1, currently receiving radiation to target area/chemotherapy, gangrene in any portion of the foot with the index ulcer, planned revascularization or under evaluation for revascularization of the index limb for advanced ischemia within the next 4 weeks of Visit 1, severe limb ischemia, and any concomitant medical or psychiatric condition that would compromise the participant's ability to safely complete the study.

Enrollment began in June 2023 and the MP plans to enrol up to 5000 participants. Results presented in this paper are obtained from the analysis of the 419 eligible participants enrolled as of January 8, 2025.

### 2.2 C‐Myc Multisite Study

The DFC c‐Myc study is a multisite observational cohort study that enrolled patients with DFUs to develop and validate potential tissue‐based biomarkers (c‐Myc and p‐GR) for the prediction of complete wound healing at 12 weeks [5]. The c‐Myc study is registered at ClinicalTrials.gov ID NCT04591691. The study was conducted in accordance with the Declaration of Helsinki and was approved by the WCG (formerly Western) Institutional Review Board. All participants signed a written informed consent permitting collection and use of data and tissue samples for the study and any ancillary analyses. Eligible participants received SOC treatment which included wound cleansing, debridement, and offloading. The c‐Myc study included protocol‐mandated total contact casting and required sharp debridement to obtain tissue samples [5]. Participants were seen in clinics weekly for up to 12 weeks or until complete wound healing. One final assessment at 2 weeks after wound healing was conducted to confirm healing. A participant was considered to have completed the study if they completed the last visit (Week 12) with achieving complete healing of their DFU (with confirmation 2 weeks later) or without achieving complete healing outcome, withdrawal due to amputation, wound infection, or receiving advanced wound care. HbA1c and comorbidities for c‐Myc participants were ascertained in the same way as for MP participants.

Eligible participants were adults 18 years and older with diabetes and a Wagner grade 1–2 DFU that was 0.5–12 cm [2] in area, wound duration of at least 4 weeks, and wound area reduction over the preceding 4 weeks of < 50%. Additional inclusion criteria included glycated haemoglobin below 12%, and adequate circulation to the foot as defined by at least 2 of the following: (1) ankle brachial index (ABI) ≥ 0.6 with Doppler Waveforms, (2) absolute ankle pressure ≥ 70 mmHg, (3) toe pressure ≥ 40 mmHg, or (4) TcPO2 ≥ 40 mmHg. Exclusion criteria included evidence of Charcot's foot, osteomyelitis, connective tissue disease, active or recent malignancy (other than non‐melanoma skin cancer), receiving immune‐modulating medications, or receiving advanced adjunct wound therapy in the 30 days before enrolment.

The c‐Myc study enrolment was prematurely terminated by the National Institute of Diabetes and Digestive and Kidney Diseases (NIDDK) based on the NIDDK Observational Safety and Data Monitoring (OSMB) Committee recommendation, owing to insufficient enrolment and lack of a positive power for c‐Myc and p‐GR biomarkers in healing prediction. However, because their wound healing rates were captured in an identical fashion as in the MP study, all 140 c‐Myc participants were allowed to complete their planned study follow‐up and are included in the analysis of this paper.

### 2.3 Wound Measurements

Wound area and depth were measured after debridement using a SOC method, which involves a ruler to measure the width, length, and depth of the wound. The wound area is calculated as the product of the width and length and reported in cm [2]. Wound images were also obtained at each visit in the MP and c‐Myc studies. If debridement is performed, wound images were obtained pre‐ and post‐debridement. Analyses for both c‐Myc and MP studies use the SOC measurements for wound area and wound depth; however, if SOC images were not available, then surface area measured by a 3‐dimensional digital imaging system (InSight, eKare Inc., Fairfax VA) after debridement was used due to a strong correlation between these two types of area measurements [5]. In the MP, eKare measurements were used for 6% of participants (25 out of 419) and in c‐Myc, they were used for 2% of participants (3 out of 140), due to the unavailability of SOC measurements.

### 2.4 Wound Duration

At their baseline visit, participants were asked how long they had the index DFU, and their response was recorded in weeks.

### 2.5 Study Outcomes

The primary outcome was healing status, defined as complete wound closure with skin re‐epithelialization confirmed by the provider at two consecutive study visits 2 weeks apart [12]. Healing status was assessed at each visit and categorised as healed by week X, or non‐healed. Once classified as healed, a wound was considered healed at all subsequent visits up to 32 weeks, defining healing as a terminal outcome for this analysis. Amputations involving the index ulcer were treated as non‐healed, defining amputation as a terminal outcome. Non‐healed wounds represented a non‐terminal outcome. Healing outcomes at a specific time point (e.g., week X) were considered missing (right‐censored) when the participant was lost to follow‐up, died, or had not yet attended the week X visit and remained unhealed at their most recent assessment. For intermittent missing visits followed by a recorded healing status, missing outcomes were treated as non‐healed.

### 2.6 Statistical Analysis

#### 2.6.1 Descriptive Baseline Characteristics

We report the baseline cohort characteristics of study participants separately for MP and c‐Myc, which include age at consent (years), sex, race, ethnicity, HbA1c (%), baseline infection, comorbidities, baseline wound area (cm [2]), wound duration (weeks) and baseline wound depth (in cm).

#### 2.6.2 Estimation of Healing Rates and Amputation Rates

We estimate cumulative healing rates at fixed time points—weeks 4, 8, 12, 16, 20, 24, 28 and 32—corresponding to the MP visit schedule. At each time point, participants are labelled into one of the three mutually exclusive wound states according to the study's outcome definitions: (I) Healed (terminal), (II) Amputation (terminal), or (III) Non‐healed. Participants in the Non‐healed state can transition to Healed, transition to Amputation, or remain Non‐healed at the subsequent visit. To account for missing (right‐censored) outcomes and the presence of Amputation as a competing risk for Healing, we use time‐to‐event analysis and estimate cumulative healing rates at each time point using the Aalen‐Johansen estimator [13], an extension of the Kaplan–Meier estimator that accounts for multi‐state scenarios. Collectively, these estimates form the healing trajectory, defined as the cumulative probability of healing over time. The corresponding time‐specific confidence intervals (95%) are calculated using the Greenwood‐type recursion formula [14]. Such analyses are performed separately for the c‐Myc study, focusing on 1‐week intervals up to week 12 due to its shorter study duration. Time‐specific cumulative amputation rates are estimated similarly. The results from the MP and c‐Myc studies are compared with previously published rates reported by Margolis et al. (1999, 2003 and 2022) [7, 8, 9] and Coye et al. [10]. These historical benchmarks represent cross‐sectional rates derived from complete‐case analyses at a few isolated time points, whereas our analysis utilises time‐to‐event methods to account for censoring and competing risks, modeling the healing trajectory across the entire study timeline.

#### 2.6.3 Baseline Models for Prediction of Healing

A logistic regression model is used to predict healing outcome by week X with wound characteristics, including baseline wound area (cm [2]) and baseline wound duration (weeks), both being log‐transformed to correct their skewness as done in previous studies [8, 9]. Models were fit using complete‐case data on baseline wound area and duration. Recorded values of zero for either covariate were treated as missing, as they likely reflect measurement or data‐entry error. Denoting p_X_ = Prob [Wound is healed by *X* weeks], we use the following logistic model:

(1) $$\text{logit}\;\left(p_{X}\right)=\beta_{0}+\beta_{1}\;\log\;\left(\text{Wound Duration}\right)+\beta_{2}\;\log\;\left(\text{Wound Area}\right),$$

where *X* varies over 4, 8, 12, 16, 20, 24, 28, and 32 weeks aligned with the MP visit schedule. The amputations are treated as non‐healed. For the completed c‐Myc study, we adopt a complete case analysis at each week under a missing‐at‐random (MAR) assumption. For the ongoing MP study, the MAR assumption is likely violated due to differential follow‐up—participants with earlier healing or amputation are more likely to have complete data at later time points. To correct the resulting bias, we apply inverse probability weighting (IPW) within the logistic model. IPW adjusts the contribution of each participant to the model by assigning each participant a weight according to the inverse of their estimated probability of having complete data at a given week. These probabilities are computed based on the observed outcome trajectory of that participant (Data S1). This approach properly downweighs participants with early terminal outcomes relative to those who remained non‐healed longer, ensuring that the model accurately reflects the entire study population by accounting for differential follow‐up. Coefficient estimates of the model parameters are reported as a function of weeks of care, together with pointwise 95% confidence intervals. The predictive performance at each week *X* is evaluated using AUC (area under the receiver operating characteristic curve), with 95% confidence intervals constructed using a bootstrap procedure with 2000 replicates. The analyses are performed separately for the MP and c‐Myc studies.

A sensitivity analysis was conducted by repeating the above analyses after removing extreme outliers in wound duration or wound area, formally defined as values exceeding the interquartile range (IQR) by a factor of three relative to the upper or lower quartiles (Data S2). Additionally, we added log‐transformed wound depth to the logistic model (1) to evaluate its potential effect on the probability of healing (Data S3). We additionally assessed sensitivity to the handling of zero values by repeating the analyses with zeros replaced by a small constant prior to log‐transformation.

#### 2.6.4 Sample Size and Power Considerations for Clinical Trial Design

The goal of the DFC is to leverage the findings from the biomarker studies to inform clinical trials. Planning of clinical trials for DFUs requires careful consideration of sample size to ensure adequate statistical power, with the effect size potentially differing by trial duration. Several recent Phase III clinical trials compare an experimental (active) treatment relative to a control group (often usual SOC or placebo), randomising participants 1:1 to either group, with the primary efficacy endpoint of complete healing (as described above) at 10 to 12 weeks, and effect size (absolute difference between active treatment and control group healing rates) of 20% [15, 16, 17, 18]. Using the range of estimated healing rates from the MP study at various time points, we calculated the required sample size to achieve at least 80% power to detect various treatment effect sizes using a two‐sided Type I error of 0.05. We used a normal approximation of the likelihood ratio test of two binomial proportions to calculate the sample size (PASS 2024 Power Analysis and Sample Size Software (2024). NCSS LLC. Kaysville, Utah, USA).

Because wound characteristics may influence healing rates, we also examined how predicted healing rates vary as a function of baseline wound area and wound duration. Predicted probabilities from model (1) were evaluated at the 1st quartile, median and 3rd quartile values of these variables in the MP dataset. This analysis was used to assess how healing rates—and therefore statistical power—might vary across subgroups, informing potential inclusion criteria and trial design.

## 3 Results

### 3.1 Descriptive Baseline Characteristics

Table 1 the baseline characteristics of study participants, stratified by study (MP and c‐Myc). Across both studies, most participants were male (77.1% in MP, 75.7% in c‐Myc) and had similar median ages (59.3 in MP, 57.6 years in c‐Myc). Median HbA1c levels at baseline were higher in the c‐Myc (8.0%) compared to the MP (7.4%). The race and ethnicity were similar across studies, characterised by a majority of participants identifying as White (> 70%). Both studies also included substantial representation of Black participants (> 17%) and Hispanic participants (> 14%).

**TABLE 1:** Baseline characteristics of the study participants, separately for MP and c‐Myc studies.

Characteristic	MP	c‐Myc
*N* (Patients)	419	140
Age at consent (years)		
*n* (%)	419 (100.0%)	140 (100.0%)
Mean (SD)	58.9 (11.3)	56.7 (10.6)
Median (Q1, Q3)	59.3 (52.0, 66.7)	57.6 (50.6, 64.2)
Min, Max	27.3, 93.6	29.8, 81.0
Sex		
*n* (%)	419 (100.0%)	140 (100.0%)
Female	95 (22.7%)	34 (24.3%)
Male	324 (77.3%)	106 (75.7%)
Race		
*n* (%)	419 (100.0%)	140 (100.0%)
American Indian or Alaska Native	21 (5.0%)	4 (2.9%)
Asian	5 (1.2%)	3 (2.1%)
Black or African‐American	72 (17.2%)	26 (18.6%)
Native Hawaiian or Other Pacific Islander	1 (0.2%)	1 (0.7%)
White	300 (71.6%)	102 (72.9%)
Prefer not to answer	20 (4.8%)	5 (3.6%)
Ethnicity		
*n* (%)	418 (99.8%)	140 (100.0%)
Hispanic or Latino	81 (19.4%)	20 (14.3%)
Not Hispanic or Latino	328 (78.5%)	117 (83.6%)
Prefer not to answer	9 (2.2%)	3 (2.1%)
HbA1c (%)		
*n* (%)	366 (87.4%)	137 (97.9%)
Mean (SD)	8.0 (2.2)	8.4 (2.1)
Median (Q1, Q3)	7.4 (6.5, 9.0)	8.0 (6.8, 9.6)
Min, Max	4.3, 15.7	4.4, 15.0
Wound area (cm2)		
*n* (%)	400 (95.5%)	140 (100.0%)
Mean (SD)	7.4 (14.1)	2.8 (2.9)
Median (Q1, Q3)	2.2 (0.7, 7.0)	1.5 (1.0, 3.8)
Min, Max	0.0, 125.0	0.4, 15.2
Wound duration (weeks)		
*n* (%)	418 (99.8%)	140 (100.0%)
Mean (SD)	46.53 (113.9)	36.7 (64.4)
Median (Q1, Q3)	16.9 (8.3, 43.8)	16.0 (6.0, 38.0)
Min, Max	0.7, 1695.2	0.0, 500.0
Wound depth (cm)		
*n* (%)	395 (94.3%)	132 (94.3%)
Mean (SD)	0.4 (0.5)	0.4 (0.5)
Median (Q1, Q3)	0.2 (0.1, 0.4)	0.3 (0.2, 0.5)
Min, Max	0.0, 3.5	0.0, 4.0
Baseline infection (WIfI Classification)		
*n* (%)	418 (99.8%)	140 (100.0%)
Uninfected	351 (84%)	132 (94.3%)
Infected	67 (16%)	8 (5.7%)
Nephropathy/CKD		
*n* (%)	418 (99.8%)	140 (100.0%)
No	231 (55.3%)	91 (65%)
Yes	187 (44.7%)	49 (35.0%)
Retinopathy		
*n* (%)	419 (100.0%)	140 (100.0%)
No	275 (65.6%)	94 (67.1%)
Yes	144 (34.4%)	46 (32.9%)
Coronary artery disease		
*n* (%)	419 (100.0%)	140 (100.0%)
No	316 (75.4%)	114 (81.4%)
Yes	103 (24.6%)	26 (18.6%)

*Note:* Summary statistics include mean (standard deviation), median (interquartile range, IQR), and observed range (min, max) for continuous variables, and counts with percentages for categorical variables. For each variable, the number of non‐missing values n is reported as count (with percentages in brackets).

Abbreviations: IQR, interquartile range; MP, Open Wound Master Protocol; SD, standard deviation.

Wound characteristics notably differed between the two studies, attributed to the differences in the inclusion criteria. MP participants presented with significantly larger wounds at baseline, with a median area of 2.2 cm [2] (IQR: 0.7–7.0 cm [2]) compared to 1.5 cm [2] (IQR: 1.0–3.8 cm [2]) in the c‐Myc participants. Median wound duration was similar across the two studies (MP: 16.9 weeks [IQR: 8.3–43.8 weeks], c‐Myc: 16.0 weeks [IQR: 6.0–38.0 weeks]), although in the MP study there was a more pronounced skew towards long‐duration wounds. Median wound depth was also similar, indicating shallow to moderate wounds (median depth ≤ 1 cm in both studies).

Additionally, the two studies differed in baseline infection rates (with higher rates in MP compared to c‐Myc), and the rates of comorbidities. MP participants had higher rates of nephropathy and coronary artery disease compared to c‐Myc participants, while retinopathy rates were comparable between studies.

### 3.2 Estimation of Healing Rates and Amputation Rates

**FIGURE 1:**
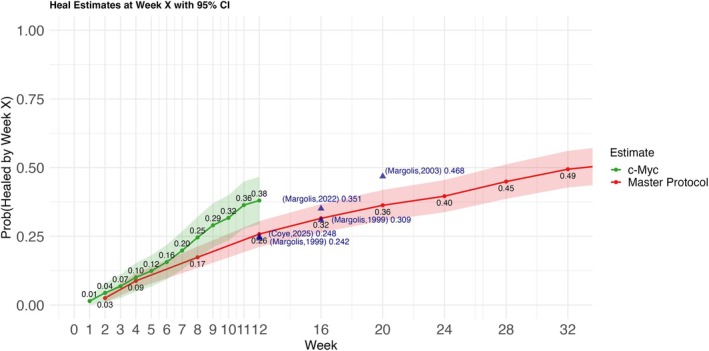
Estimated cumulative healing rates with 95% confidence intervals by week for MP (red) and c‐Myc (green) studies. Estimated healing rates from previous studies (12 week and 20 weeks from Margolis, 1999 [7]; 20 week from Margolis, 2003 [8]; 16 week from Margolis, 2022 [9]; and 12 week and 20 week from Coye, 2025) [10] are included for comparison.

Figure 1 presents the time‐course of estimated healing rates (healing trajectory) with pointwise 95% confidence intervals (CIs) for MP and c‐Myc studies. In MP, approximately 26% of participants (95% CI: 21%–31%) had DFUs heal by week 12, increasing to around 49% by week 32 (95% CI: 43%–56%). In c‐Myc (Figure 1), around 38% (95% CI: 29%–47%) of participants healed by week 12. The increase in healing rate from weeks 2 to 12 was more rapid in c‐Myc compared to the MP participants, although this difference should be interpreted in the context of differences in study design and care delivery, including more frequent research visits and protocolised offloading and debridement in the c‐Myc study.

**FIGURE 2:**
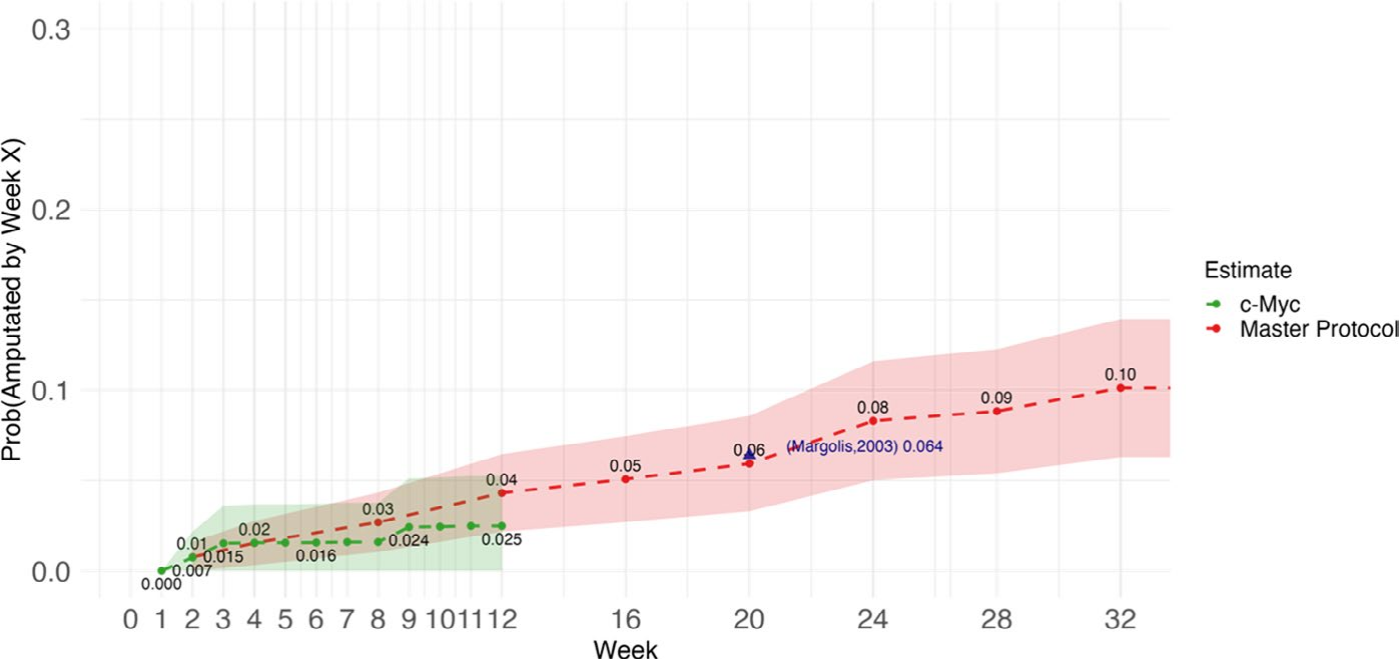
Estimated cumulative amputation rates (with 95% CI) are shown for the MP (red) and c‐Myc (green) studies. An estimate from a previous study (at 20 weeks, Margolis, 2003) [8] is included for comparison.

Figure 2 presents the time‐course of estimated amputation rates for MP and c‐Myc studies. In MP, amputation rates increase from 4.3% (95% CI: 2.2%–6.5%) by week 12 to 10% (95% CI: 6.3%–14%) by week 32. In c‐Myc, the amputation rate by week 12 was only 2.5% (95% CI: 0%–5.2%) due to only three amputation events reported over the 12‐week follow‐up.

### 3.3 Baseline Model for Prediction of Healing

**FIGURE 3:**
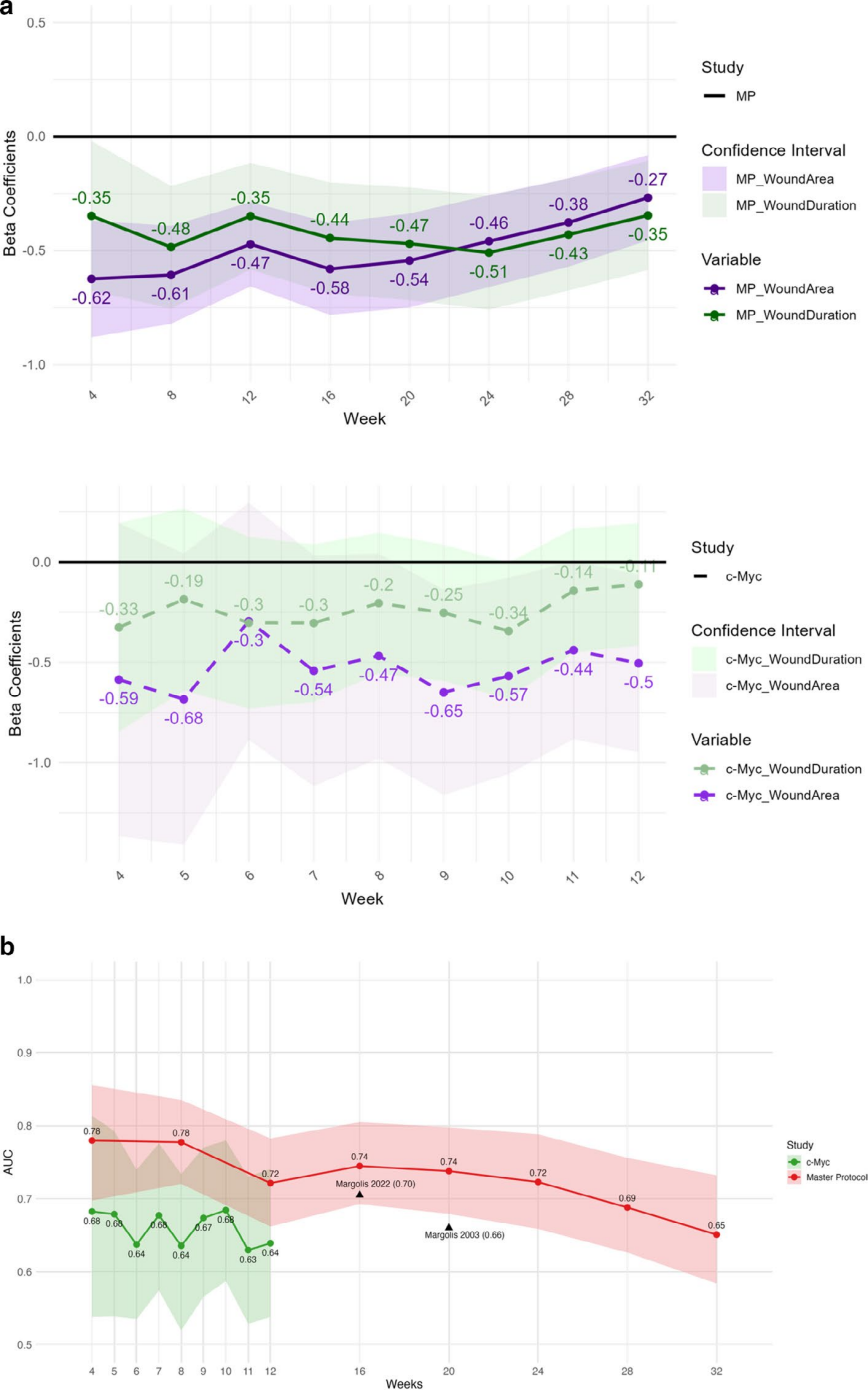
(a) Beta coefficient estimates (±95% confidence intervals) for baseline wound duration (green with shade) and baseline wound area (purple with shade) from logistic regression models predicting the probability of wound healing over time, stratified by the MP (top panel, solid, darker) and c‐Myc (bottom panel, dashed, lighter) studies. (b) The predictive performance (area under the receiver operating characteristic curve, AUC, with 95% bootstrap confidence interval) of the logistic regression model for the probability of wound healing by week for the MP (red) and c‐Myc (green) studies using baseline wound area and duration. Previous AUC estimates at 20 weeks in Margolis, 2003 [8] and at 16 weeks in Margolis, 2022 [9] are included for comparison.

Figure 3a displays the estimated coefficients from model (1) for log‐wound duration and log‐wound area as a function of time, along with pointwise 95% confidence intervals, presented separately for MP and c‐Myc. In MP, both longer wound duration and larger wound area were associated with a lower probability of healing (estimated coefficients *β* < 0 for each predictor) and were statistically significant (*p* < 0.05) at all weeks 4, 8, 12, 16, 20, 24, 28 and 32. The magnitude of association for wound duration remained relatively stable throughout the 32‐week follow‐up, with coefficients ranging from −0.51 to −0.35. Wound area had initially a stronger negative association than duration through week 20, after which the two effects became more comparable. In c‐Myc, both coefficients were also negative; however, only the wound area showed a significant association with healing from week 9 onward. The strength of association for wound duration was weaker and did not reach statistical significance at the 0.05 level.

Figure 3b illustrates the AUC values from the prediction model (1) for both MP and c‐Myc studies, with 95% bootstrap confidence intervals. For MP, the model demonstrates strong predictive accuracy from week 4 to 24 with AUC values consistently at or above 0.72, peaking around 0.78 in early weeks. From week 28 onward, AUC drops below 0.7, suggesting a modest decline in predictive performance that may be due to a smaller sample size at later follow‐up time points. These findings are similar to those reported in previous studies [8, 9], reaffirming that both wound duration and wound area are strong predictors for the chance of wound healing. For c‐Myc, AUC values are smaller and stay below 0.7, albeit the confidence intervals overlap. These results are also presented in table form in Table S1.

In sensitivity analyses, excluding outliers had minimal impact on the resulting AUC, with estimated coefficients being similar in magnitude and statistically significant, confirming the robustness of these results (Table S2). The addition of wound depth as a predictor did not further improve AUC, and wound depth was not significant after adjusting for both wound area and duration in the prediction model (Table S3). Only one MP participant had a recorded wound area of 0 cm [2], and 1 c‐Myc participant had a recorded wound duration of 0 weeks; replacing these zero values with a small constant rather than treating them as missing produced negligible changes in coefficient estimates and identical AUC values.

### 3.4 Sample Size and Power Considerations for Clinical Trial Design

Table 2 presents the calculated sample sizes for effect sizes for a given intervention ranging from 5% to 25%, assuming that 20% to 60% of participants in the control arm achieve healing, with a 5% two‐sided Type I error and 80% power. The range of total sample sizes ranges from more than 3000 (to detect treatment differences of 5%) to 98 (to detect larger treatment differences of 25%). Within a given effect size, the sample size is also influenced by the healing rate in the control arm. Our estimated healing rates from the MP range from 26% at week 12 to 49% at week 32. Using a 20% effect size with these two estimates, the sample size is approximately 178 and 186, respectively. Using smaller effect sizes, the difference in sample size is larger between estimated healing rates in the control group of 25% and 50% (e.g., for a 10% treatment difference, the sample size is 658 versus 776, respectively—an 18% increase).

**TABLE 2:** Sample size required for 2‐arm clinical trial with 80% power and 2‐sided Type I error of 5% as a function of healing rate in control group and effect size.

% Participants achieving complete healing			*n*/group	Total *N*
Active treatment group	Control group	Effect size*		
0.25	0.20	0.05	1094	2188
0.30	0.25	0.05	1251	2502
0.35	0.30	0.05	1377	2754
0.40	0.35	0.05	1471	2942
0.45	0.40	0.05	1534	3068
0.50	0.45	0.05	1565	3130
0.55	0.50	0.05	1565	3130
0.60	0.55	0.05	1534	3068
0.65	0.60	0.05	1471	2942
0.30	0.20	0.10	294	588
0.35	0.25	0.10	329	658
0.40	0.30	0.10	356	712
0.45	0.35	0.10	376	752
0.50	0.40	0.10	388	776
0.55	0.45	0.10	392	784
0.60	0.50	0.10	388	776
0.65	0.55	0.10	376	752
0.70	0.60	0.10	356	712
0.35	0.20	0.15	138	276
0.40	0.25	0.15	152	304
0.45	0.30	0.15	163	326
0.50	0.35	0.15	170	340
0.55	0.40	0.15	173	346
0.60	0.45	0.15	173	346
0.65	0.50	0.15	170	340
0.70	0.55	0.15	163	326
0.75	0.60	0.15	152	304
0.40	0.20	0.20	82	164
0.45	0.25	0.20	89	178
0.50	0.30	0.20	93	186
0.55	0.35	0.20	96	192
0.60	0.40	0.20	97	194
0.65	0.45	0.20	96	192
0.70	0.50	0.20	93	186
0.75	0.55	0.20	89	178
0.80	0.60	0.20	82	164
0.45	0.20	0.25	54	108
0.50	0.25	0.25	58	116
0.55	0.30	0.25	61	122
0.60	0.35	0.25	62	124
0.65	0.40	0.25	62	124
0.70	0.45	0.25	61	122
0.75	0.50	0.25	58	116
0.80	0.55	0.25	54	108
0.85	0.60	0.25	49	98

*Note:* *Effect Size = Active Treatment Group Rate–Control Group Rate. The sample size was computed using PASS 2024, version 24.0.5; tests for two proportions, normal approximation method for Likelihood Ratio Test.

**FIGURE 4:**
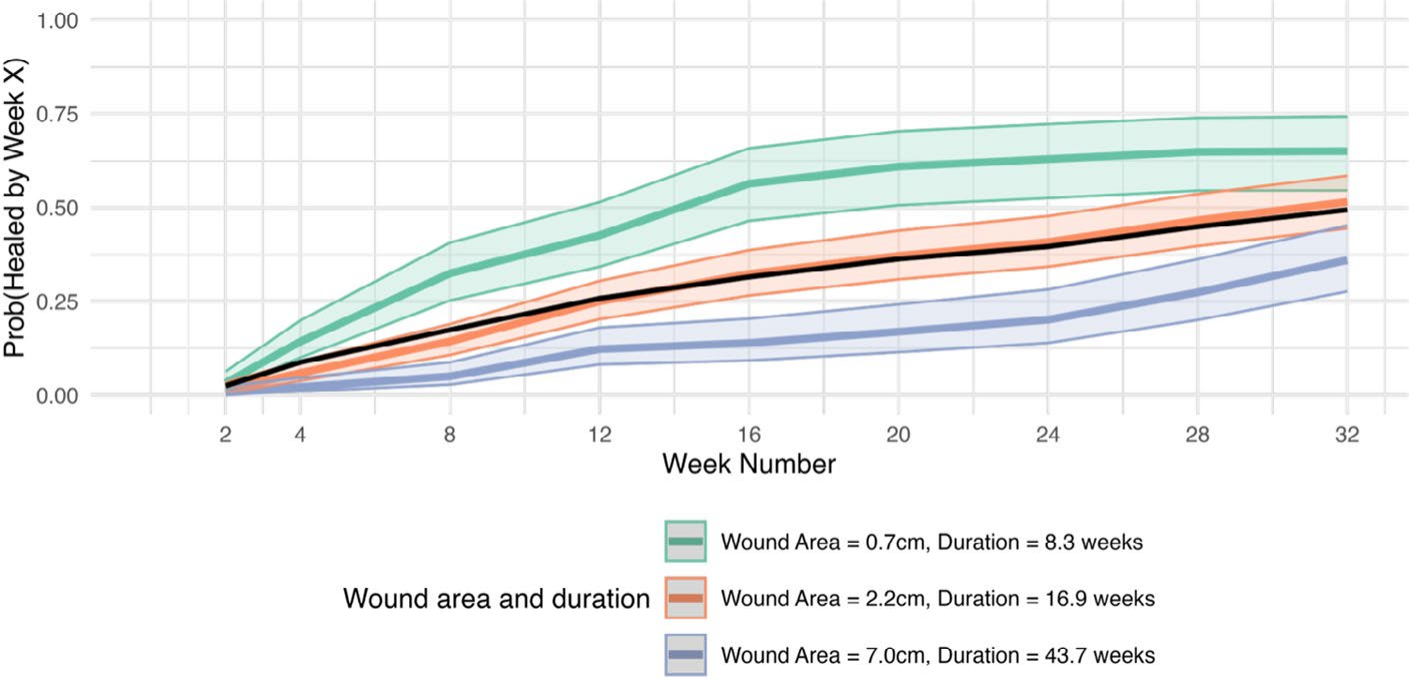
Estimated probability of healing by week X with 95% confidence interval based on logistic model (1) as a function of wound area and wound duration. The black line corresponds to the overall estimated healing rates from Figure 1, whereas green, orange, and blue correspond to the lower quartiles for wound area and duration, medians of wound area and duration, and the upper quartiles for wound area and duration based on the MP study.

Figure 4 presents the estimated time course of healing rates, with pointwise 95% confidence intervals, based on logistic model (1) fit to the MP study. The black line reflects the overall estimated healing rates shown in Figure 1, while the green, orange, and blue lines represent predicted healing rates for combinations of wound area and duration at the lower quartiles, medians, and upper quartiles, respectively, based on the distribution in the MP. The differences across these strata are substantial and consistent with model expectations: Wounds that are smaller and of shorter duration are associated with faster healing. These findings reinforce the importance of accounting for baseline wound characteristics in both study design and interpretation.

## 4 Discussion

This study is one of the first to rigorously and methodologically evaluate wound healing times in contemporary DFUs using 2 prospective observational studies from the Diabetic Foot Consortium to inform future clinical trial design. We demonstrated that the current DFUs healing time often exceeds the assumed 12‐week timelines used in prior DFU studies. We also found that, in these cohorts, only about one‐third of DFUs heal by 12 weeks while receiving the usual SOC practice. These findings suggest that assuming shorter follow‐up periods as the norm may limit true assessment of treatment effects. Importantly, we confirmed that wound characteristics—such as area and duration—significantly affect healing rates, and that depending on the magnitude of the treatment effect, these baseline healing rates can substantially influence sample size requirements, underscoring the importance of incorporating wound characteristics in the design of DFU studies. Additionally, we also demonstrated that healing rates have remained largely consistent in the last 2 decades since seminal work on wound healing rates was published by Margolis et al. in the early 2000s [7, 8]. Critically, the use of multi‐site data and broad inclusion criteria (MP study) ensures our findings have high generalizability by capturing broad patient representation across demographic and wound characteristics, as well as reflecting real‐world heterogeneity in SOC.

Placing our findings into perspective, these data align with historical literature in this field. For instance, the healing rates we observed at weeks 12 and 16 are in line with previously reported 12‐week rates from Margolis et al. [7] and Coye et al. (2025) [10], and 16‐week rate from Margolis et al. (2022) [9]. Similarly, the amputation rate we observed at week 20 is consistent with the 20‐week rate reported by Margolis et al. [8]. Although our estimated healing rate at week 20 is higher than the 20‐week rate reported in Margolis et al. [7], it is lower than more recent 20‐week rates reported in Margolis et al. (2003) [8] and Coye et al. [10].

Wound duration consistently emerged as a strong predictor of healing in DFU despite ongoing debates regarding its exact clinical significance and reliability. Although some have questioned whether wound duration is a true reflection of healing potential or simply a surrogate marker for other underlying factors, our findings reaffirm that longer wound durations are associated with poorer healing outcomes. This aligns with previous studies, including those by Margolis et al. [8, 9], where extended wound durations were consistently linked to decreased healing rates. While the interpretation of wound duration may vary, its strong association with healing in this study supports its role as a valuable clinical predictor. However, wound duration alone cannot fully explain the complexities of DFU healing. Future research should explore its relationship with other factors such as patient comorbidities and wound therapy history.

Second, wound characteristics varied substantially between the two studies analysed, MP and c‐Myc, with MP participants presenting significantly larger baseline wound areas. Logistic regression modeling showed that predicted healing rates at 12 to 24 weeks can differ by 30%–40% depending on wound size and duration, which helps explain the observed differences in healing outcomes. This variability is critical because enrolling only patients with small, short‐duration wounds would result in substantially higher healing rates, thereby affecting required sample sizes. However, restricting enrolment based on wound characteristics may prolong trial duration, as fewer patients would meet the eligibility criteria.

When comparing healing trajectories across studies, several design elements should be noted. Although both studies enrolled participants receiving standard of care treatment, the care models differed meaningfully. The MP study followed participants for up to 32 weeks in the present analysis with biweekly visits initially and monthly thereafter, and reflected heterogeneous usual care, with offloading, debridement, and wound management performed at the clinician's discretion. In contrast, c‐Myc participants were seen weekly for up to 12 weeks, and received a more protocolized care pathway, including protocol‐mandated total contact casting and required excisional sharp debridement of the wound edges to obtain tissue samples. These differences in visit frequency, offloading, debridement, and overall treatment intensity may partially explain the faster early healing observed in c‐Myc and should be considered when interpreting cross‐study comparisons. Higher rates of nephropathy and coronary artery disease in MP relative to c‐Myc are consistent with a more heterogeneous patient population in MP and may also contribute to the differences in healing trajectories between the two studies. Therefore, differences in healing trajectories between MP and c‐Myc should not be attributed solely to baseline wound area and wound duration. The smaller c‐Myc sample, partly reflecting early termination of enrollment, also contributes to reduced power and wider confidence intervals relative to MP. Direct comparisons of healing rates between MP and c‐Myc should therefore be made cautiously, as the populations and care protocols are not fully equivalent.

The same cautions should be applied when comparing the healing rates presented here with other rates reported in the literature. Specifically, the rates reported in Margolis et al. [7] and Coye et al. [10] are based on a meta‐analysis of the control arms of clinical trials that follow the SOC. These control arms are heterogeneous in the choice of SOC treatment (e.g., dressing and debridement schedule) and excluded participants with baseline wound infections. Given that 16% of MP participants presented with a baseline infection, this difference in inclusion criteria may explain why we observe lower 20‐week healing rates compared to those reported by Coye et al. [10]. The rates reported in Margolis et al. [8] and Margolis et al. [9] are based on multi‐centre cohort studies with broad inclusion criteria and broader use of SOC, making them more similar to the MP study. However, the variability in treatment choices and patient populations across centres means there is still no one‐to‐one comparison. The study differences highlight how treatment factors and patient inclusion criteria can further influence outcomes. Further, both MP and c‐Myc studies had predominantly male and predominantly White participants. Male predominance is consistent with epidemiological data showing higher DFU prevalence in males [19], although reported male proportions vary considerably across prior DFU studies (~54% in Margolis et al. [8], ~73% in Margolis et al.) [9], placing our studies at the upper end of this range. These demographic patterns may limit external validity, and our findings should be generalised with caution to settings serving more diverse populations. From a methodological perspective, these historical benchmarks represent cross‐sectional rates derived from complete‐case analyses at a few isolated time points, whereas our analysis utilises time‐to‐event methods to account for censoring and competing risks, modeling the healing trajectory across the entire study timeline. Taken together, these findings emphasise the importance of accounting for wound characteristics, management protocols when designing clinical trials and underscore the challenge of generalising results across heterogeneous populations of patients with DFUs.

Interestingly, wound depth did not prove to be a significant predictor of healing in this study, which is consistent with qualitative observations but was not previously quantified. Although one might expect wound depth to play a key role in healing outcomes, it appears to be less informative than initially anticipated. In the MP, wound depth was measured using a standardised ruler‐based method after debridement. More advanced imaging technologies could improve depth assessment, but such approaches are not currently feasible for widespread implementation in the MP study. Despite our unified approach, the finding that depth does not predict (eventual) healing is not entirely surprising. Wound depth can be notoriously difficult to measure accurately, often leading to significant measurement errors. Variations in clinical techniques, observer discrepancies, and challenges in imaging methods could all contribute to the variability in depth assessments. Given these limitations, it may be prudent to focus on more reliable and clinically relevant factors, such as wound area and duration, when evaluating the potential for healing in DFUs as we have within our models.

Despite decades of research, healing and amputation rates for DFUs have shown little improvement over the past 20 years. Healing rates in this study were consistent with historical data, and amputation rates followed similar trajectories, suggesting that current treatment approaches have reached a plateau. This stagnation underscores the urgent need for renewed efforts in diabetic foot care, particularly through the identification and incorporation of novel biomarkers and therapeutic targets. Biomarker discovery could provide crucial insights into wound healing mechanisms and help tailor treatments more effectively. Additionally, despite the established importance of wound area and duration, it appears that the most predictive window for healing occurs between weeks 12–20, highlighting this time frame as potentially critical for intervention strategies. This suggests that future studies should consider extending follow‐up beyond 12 weeks to better capture treatment effects and distinguish healers from non‐healers, while balancing sample size and trial feasibility. Our power analysis for sample size for clinical trials indicates that only relatively modest numbers of participants are needed for clinically meaningful interventions (20% effect size).

While our analysis focused on complete wound healing as the primary outcome, future research may benefit from evaluating surrogate endpoints such as early reductions in wound area, which could serve as an intermediate indicator of healing. While not assessed in this study, these measures could provide a timelier reflection of treatment response and may reduce the need for extended follow‐up to observe complete wound closure—the most common endpoint in DFU trials. Early clinical surrogate markers, such as ≥ 50% percent wound surface area reduction (PAR) at 4 weeks, have been shown to strongly predict eventual healing [20, 21]. Longitudinal tracking of wound surface area, as measured by length by width measurements (in cm [2]), is required by Centres for Medicare & Medicaid Services for reimbursement and remains a practical biomarker derived from patient health records that is useful to guide clinical judgements and assessment of wound progression. Our predictive models provide complementary probabilities of healing over time, allowing clinicians to stratify wounds by risk. Wounds with low predicted probability or poor early PAR may warrant closer monitoring or early escalation of therapy, while those with high probability can continue SOC. The incorporation of wound area reduction as a key endpoint could streamline the evaluation of new therapies and help accelerate the approval process for promising treatments. As DFU management continues to evolve, continued refinement of outcome measures, along with the integration of emerging biomarkers, will be critical in improving trial design, patient outcomes and reducing the long‐standing burden of diabetic foot complications.

## Author Contributions

**Charlotte Xu:** study design, data analysis, results interpretation, writing – original draft, writing – review and editing. **Brian M. Schmidt:** results interpretation, writing – original draft, writing – review and editing; **Amy Krambrink:** data analyses, writing – original draft; **Junyoung Park:** study design, data analysis, results interpretation, writing – original draft; **Peter X. K. Song:** study design, results interpretation, writing – review and editing; **Teresa L. Z. Jones:** results interpretation, writing – review and editing; **Crystal M. Holmes:** writing – review and editing; **Kellen Chen:** writing – review and editing; **Wen Ye:** writing – review and editing; **Giselle Kolenic:** writing – review and editing; **Sashwati Roy:** writing – review and editing; **Chandan K. Sen:** writing – review and editing; **Rodica Pop‐Busui:** study design, results interpretation, writing – review and editing; **Cathie Spino:** conceptualization, study design, data analyses, results interpretation, writing – review and editing; **Irina Gaynanova:** conceptualization, study design, data analyses, results interpretation, writing – original draft, writing – review and editing.

## Funding

This study is conducted by the NIH‐sponsored Diabetic Foot Consortium supported through Grant IDs U01DK119100, U01DK119083, U01DK119094, U01DK119099, and U24DK122927 of the NIDDK.

## Conflicts of Interest

The authors declare no conflicts of interest.

## Supporting information


**Data S1:** Supporting Information.

## Data Availability

The data that support the findings of this study are available on request from the corresponding author. The data are not publicly available due to privacy or ethical restrictions.
