# Supplementary material for: Longitudinal Healing and Amputation Trajectories in Diabetic Foot Ulcers: Predictive Power of Wound Area and Duration and Sample‐Size Implications From the Diabetic Foot Consortium
Source: Wound Repair Regen. 2026 Jul 15;34(4):e70189. doi: 10.1111/wrr.70189 (PMC13373335; doi:10.1111/wrr.70189)
Supplement: Supplementary file 1 — Data S1: Supporting Information. [file WRR-34-0-s001.docx]

**SUPPLEMENTARY MATERIALS**

This appendix has been provided by the authors to give readers additional information about their work.

Supplement to: Xu C, Schmidt BM, Gaynanova I, et al. Profiling Longitudinal Wound Healing: Analysis of Healing Rates and the Predictive Power of Wound Area and Duration in the Diabetic Foot Consortium. 2026.

**Supplementary Materials - Table of Contents**

[**Supplement** 2](#_Toc219361125)

[Supplement 1 (S1). *Construction of weights for logistic regression to account for differential missingness over time in the Master Protocol* 2](#_Toc219361126)

[Supplement 2 (S2). *Sensitivity analysis removing wound duration and wound area outliers* 4](#_Toc219361127)

[Supplement 3 (S3). *Wound depth as an additional predictor for healing by week X* 5](#_Toc219361128)

[**Supplemental Figures** 6](#_Toc219361129)

[Figure SF1. 6](#_Toc219361130)

[Figure SF2a. 7](#_Toc219361131)

[Figure SF2b. 8](#_Toc219361132)

[Figure SF3a. 9](#_Toc219361133)

[Figure SF3b. 10](#_Toc219361134)

[**Supplemental Table** 11](#_Toc219361135)

[Table ST1. *Estimated healing rates from the main manuscript represented in the table* 11](#_Toc219361136)

[**References** 12](#_Toc219361137)

[**Supplemental Acknowledgements** 13](#_Toc219361138)

# **Supplement**

## Supplement 1 (S1). *Construction of weights for logistic regression to account for differential missingness over time in the Master Protocol*

At each week $X$, only a subset of patients in the MP have non-missing healing status, as not all have yet reached their scheduled visit at that time. However, for patients who experienced healing or amputation before week $X$, outcome status is deterministically known through week 32. This induces differential probability of missingness: patients with earlier terminal events (healing or amputation) are more likely to have observed data at week X than those without.

To address this, we estimate the probability of non-missingness at week $X$ for each patient with observed data, conditional on their trajectory up to that point. We then apply inverse probability weighting (IPW), where the inverse of the estimated probability is used as a weight in a logistic regression model (1). This approach corrects for bias due to differential missingness based on healing or amputation status and yields overall healing probabilities consistent with Kaplan-Meier curves. Weighted models are fit using the glm function in R with a quasibinomial family.

**Estimation of the probability of non-missingness at week X**

At each time up to and including $X$, the outcome is 0 (not healed), $1$ (healed), or $A$ (amputation), where both $1$ and $A$ are terminal events. Once a terminal event occurs, all subsequent values are deterministically 1 or $A$, respectively. Thus, each patient has a trajectory of outcomes $s=\{s_{1},s_{2},\ldots,s_{X}\}$. Some example trajectories with $X = 5$ are

$$s_{0}=\{0,0,0,0,0\},\quad s_{A}=\{0,A,A,A,A\},\quad s_{H}=\{0,0,0,1,1\}.$$

Suppose a trajectory contains a terminal event (healing or amputation) before time $X$. In that case, it has a higher probability of being fully observed at time $X$. To see this, let $K$ be a random variable denoting the follow-up time, with $P\left( K=x \right)$ indicating the probability of missing an outcome after time x. Then if $s^{*}=\left\{ 0,0,A,A,A \right\}$ is a full trajectory, the observed trajectory can be: $\left\{ 0 \right\}$ with probability $P\left( K=1 \right)$, $\left\{ 0,0 \right\}$ with probability $P\left( K=2 \right)$, or $\left\{ 0,0,A \right\}$ with probability $P\left( K=3 \right)$, or $\left\{ 0,0,A, A \right\}$  with probability $P\left( K=4 \right)$, and $\left\{ 0,0,A,A,A \right\}$ with probability $P\left( K=5 \right)$. Because of terminality, however, observing $\left\{ 0,0,A \right\}$  or $\left\{ 0,0,A, A \right\}$ is equivalent to observing $\left\{ 0,0,A,A,A \right\}$, and thus we get $\left\{ 0 \right\}$ with probability $P\left( K=1 \right)$, $\left\{ 0,0 \right\}$ with probability $P\left( K=2 \right)$, and $\left\{ 0,0,A,A,A \right\}$ with probability $P(K\geq5)$.

Hence, to compute the probability of observing a full trajectory $s^{*}=\{s_{1},s_{2},\ldots,s_{X}\}$, we distinguish between terminal and non-terminal trajectories. For trajectories with the terminal event (healing or amputation before $X$, i.e., at time $x^{*}<X):$

$P\left( observe full s^{*} \mid s^{*} \right)=P(K\geq x^{*})$.

However, for non-terminal trajectories (no healing or amputation before $X$):

$P\left( observe full s* \mid s* \right)=P\left( K\geq X \right)=P(K=X)$.

These formulas reflect the fact that patients with earlier terminal events are more likely to have complete trajectories by week $X$.

We estimate $P(K\geq x)$ for each $x$ from 1 to $X$ using only patients without terminal events (non-healers), assuming missing at random (MAR) within this group. Let ${NM}_{j}$ denote the event of having a non-missing outcome at time j, and assume that everyone is observed at the first time point, $P({NM}_{1}) = 1$(which is true in MP). Then

$P\left( K\geq x \right)=P\left( NM_{x} \right)=P\left( {NM}_{1} \right) \Pi_{j=2}^{x}P\left( {NM}_{j} \mid{NM}_{j-1} \right)=\Pi_{j=2}^{x}P\left( {NM}_{j} \mid{NM}_{j-1} \right)$,

where we use empirical estimates

$\hat{P}({NM}_{j}\mid{NM}_{j-1}) =\frac{\# newly missing at time x=j}{\# non-healed at time x=j-1}$.

**IPW for logistic regression**

We fit the logistic regression model (1) using weighted maximum likelihood, where each observation is weighted by the inverse of the estimated probability of non-missingness at week $X$. Concretely, for each participant *i* with an observed trajectory ${s_{i}}^{*}$at week $X$, the corresponding weight is defined as

$w_{i}=\hat{1/P\left( observe full{s_{i}}^{*}|{s_{i}}^{*} \right)}$,

where the probability in the denominator is estimated as described in the previous section. The model coefficients are then estimated as

$$\hat{\beta}=argmax\sum_{i=1}^{n} w_{i}\left[ y_{iX}\log\left( p_{iX} \right)+\left( 1-y_{iX} \right)\log\left( 1-p_{iX} \right) \right],$$

where $y_{iX}$ is equal to 1 if participant *i* has healed by week $X$ and 0 otherwise, and $p_{iX}$ denotes the probability of healing by week $X$ expressed through coefficients according to model (1).

## Supplement 2 (S2). *Sensitivity analysis removing wound duration and wound area outliers*

Exploratory data analysis (Figure SF1) revealed extreme values in wound duration (up to 13 years) and wound area (exceeding 150 cm^2^). The outliers were formally identified based on three times the interquartile range (IQR) deviations from the first quartile (Q1) and third quartile (Q3), that is, the values outside the interval [Q1 - 3 × IQR, Q3 + 3 x IQR]. This resulted in the removal of 52 subjects from the MP study and 9 subjects from the c-Myc study. Model (1) was refit with these outliers removed (still using log-transformed values as in the main analysis), and the updated coefficient estimates and values of AUC were compared with results from the full data.

Figure SF2a presents updated model coefficients for wound duration and wound area with 95% confidence intervals after removal of the outliers. Figure SF2b shows corresponding updated AUC values with 95% bootstrap confidence intervals. Outlier removal led to coefficient estimates of similar magnitude and had minimal effect on AUC values, indicating that the main model's coefficient magnitudes and predictive performance are robust to outliers and not driven by subjects with extreme values for either wound area or wound duration.

## Supplement 3 (S3). *Wound depth as an additional predictor for healing by week X*

Baseline wound depth (cm) was added to the logistic regression model (1) to evaluate its potential value in predicting wound healing. As in the main analysis, recorded values of 0 cm for wound depth were treated as missing, as they likely reflect measurement or data-entry error; this excluded 15 MP and 6 c-Myc participants, in addition to those already excluded for missing or zero values for wound area or duration. A stepwise modeling approach was used to compare three models based on changes in beta coefficients and AUC: Model A included log(Wound Area); Model B included log(Wound Area) and log(Wound Depth); Model C included log(Wound Area), log(Wound Depth), and log(Wound Duration).

Figure SF3a displays the estimated coefficients from each model with 95% confidence intervals, separately for MP and c-Myc studies. Figure SF3b illustrates corresponding AUC values over time. Wound area and duration remained significant predictors after adjusting for wound depth, which did not improve predictive performance and showed non-significant coefficients (p > 0.05). These findings were consistent across both studies. In sensitivity analyses, replacing zero depth values with a small constant or applying an offset to all values prior to log-transformation produced negligible changes; wound depth remained non-significant in both cohorts.

# **Supplemental Figures**

## Figure SF1.

Histograms and scatterplots showing the baseline distributions of wound area and wound duration for the MP (top row) and c-Myc (bottom row) studies. Both variables are right-skewed, with a small number of extreme values. Scatterplots use log-scaled axes to aid visualization.


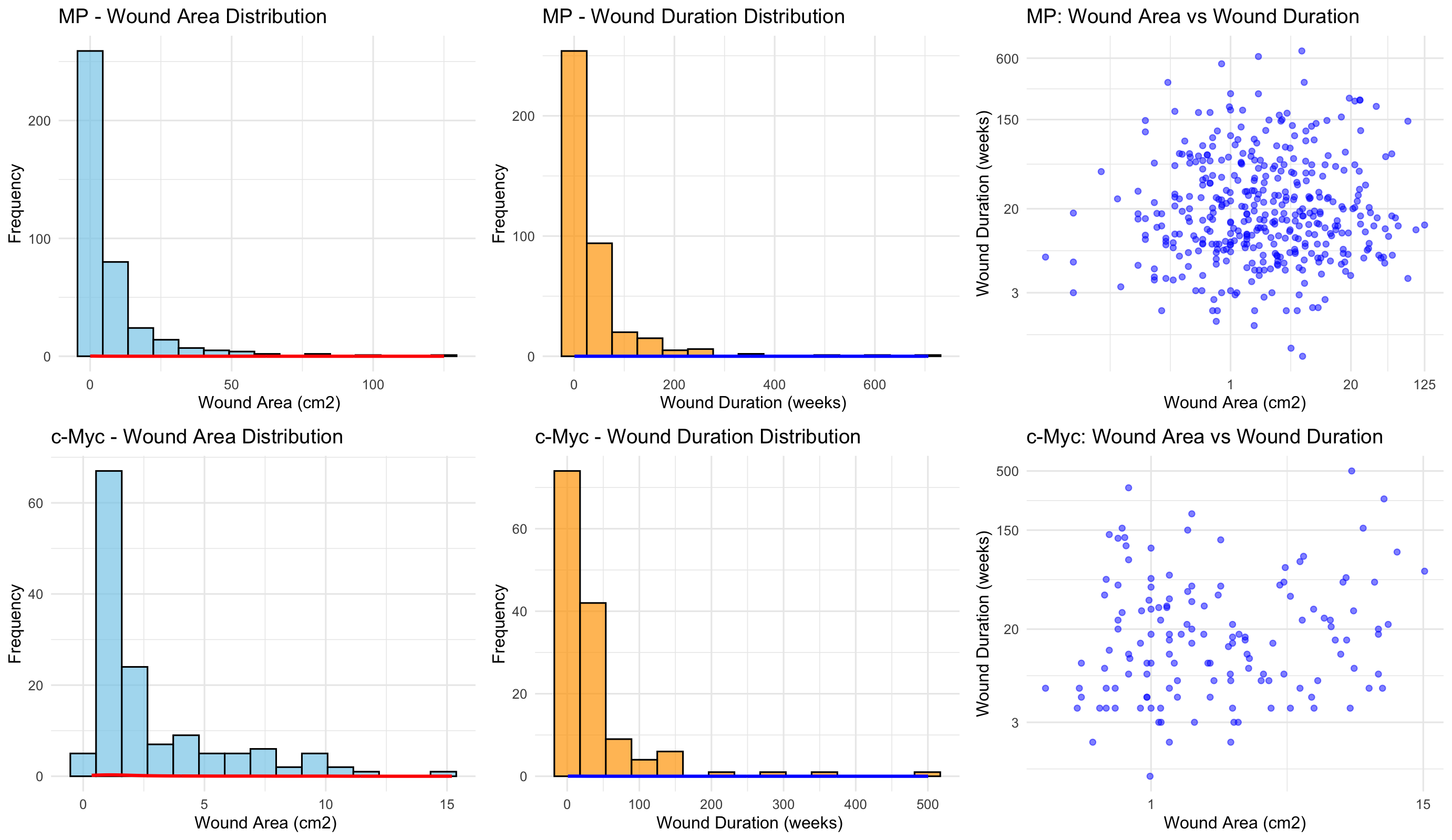


## Figure SF2a.

Beta coefficient estimates (±95% confidence intervals) for baseline wound duration (green with shade) and baseline wound area (purple with shade) from the logistic regression model (with outliers removed) predicting the probability of wound healing over time, stratified by the MP (top panel, solid, darker) and c-Myc (bottom panel, dashed, lighter) studies.


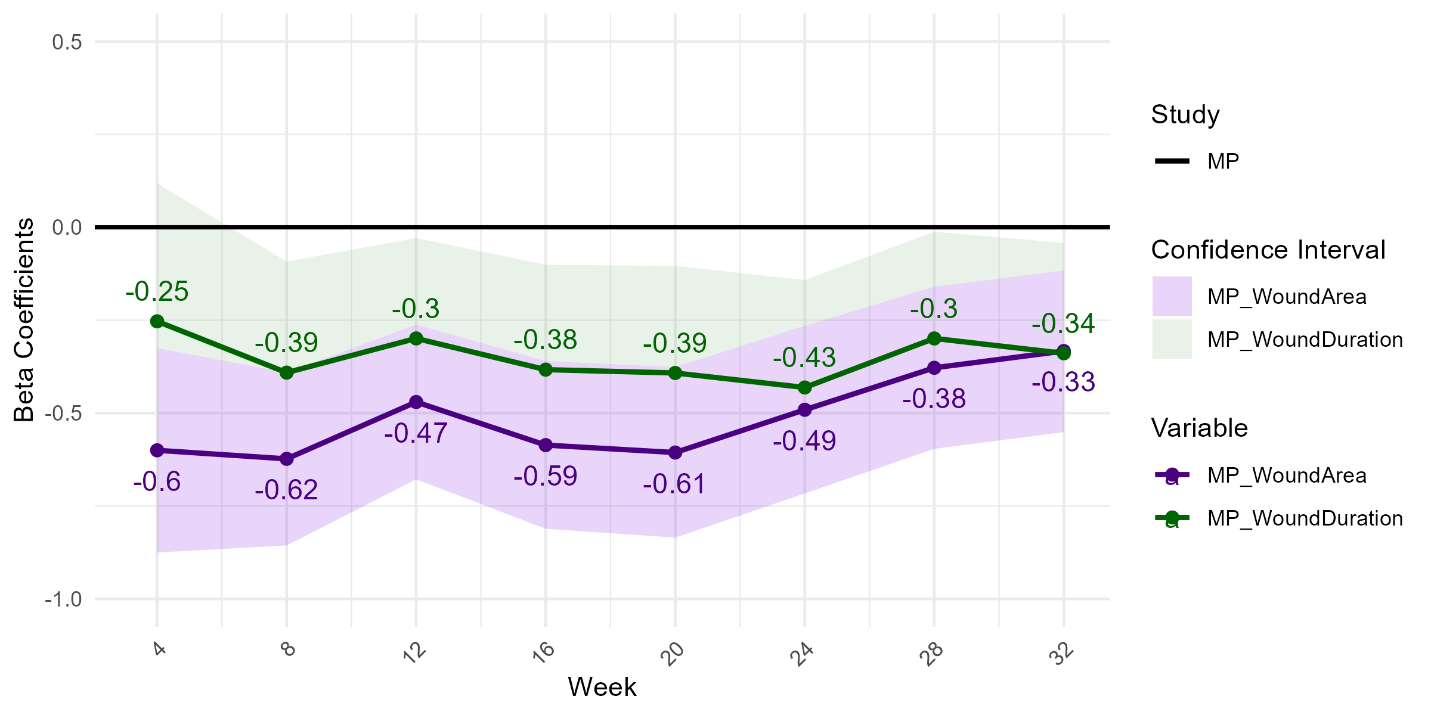


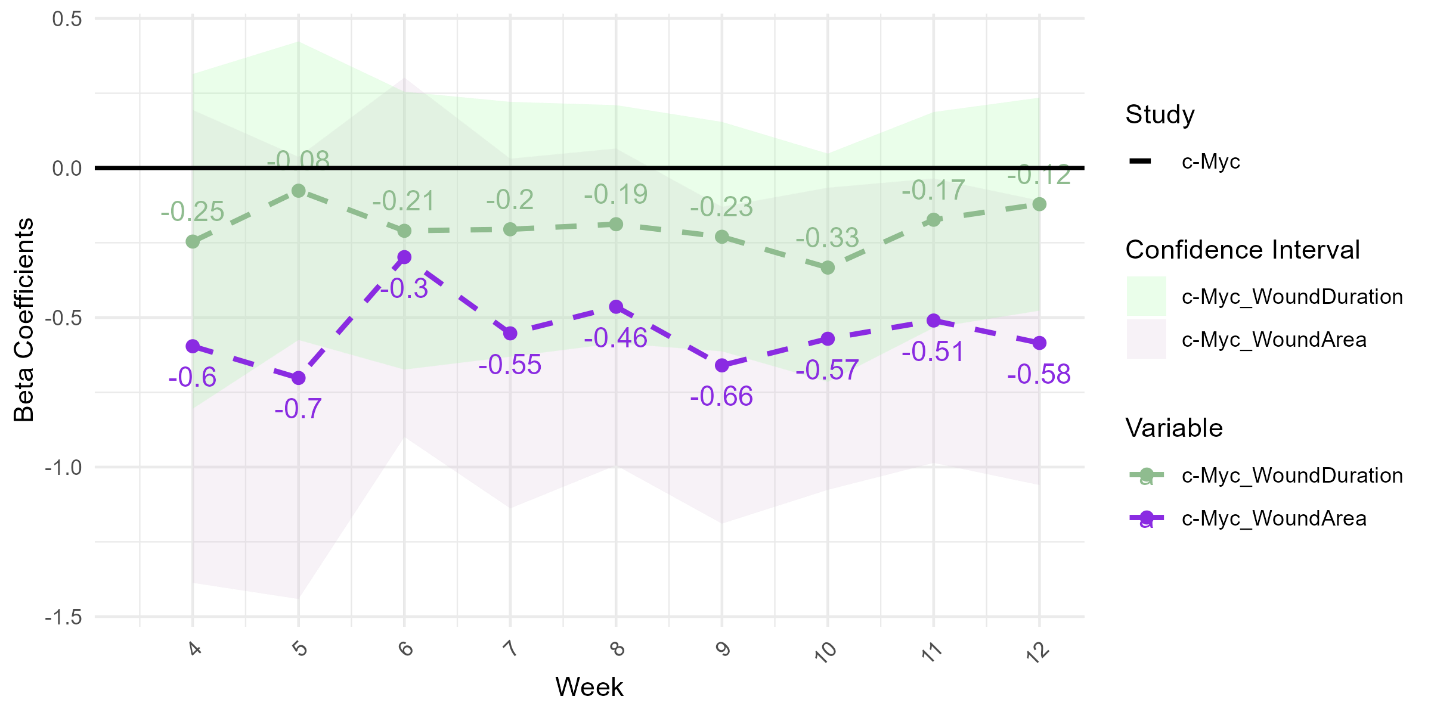


## Figure SF2b.

The predictive performance (area under the receiver operating characteristic curve, AUC, with 95% bootstrap confidence interval) of the logistic regression model (with outliers removed) for the probability of wound healing by week for the MP (red) and c-Myc (green) studies. Previous AUC estimates from Margolis, 2003^1^ and Margolis, 2022^2^ studies are included for comparison.


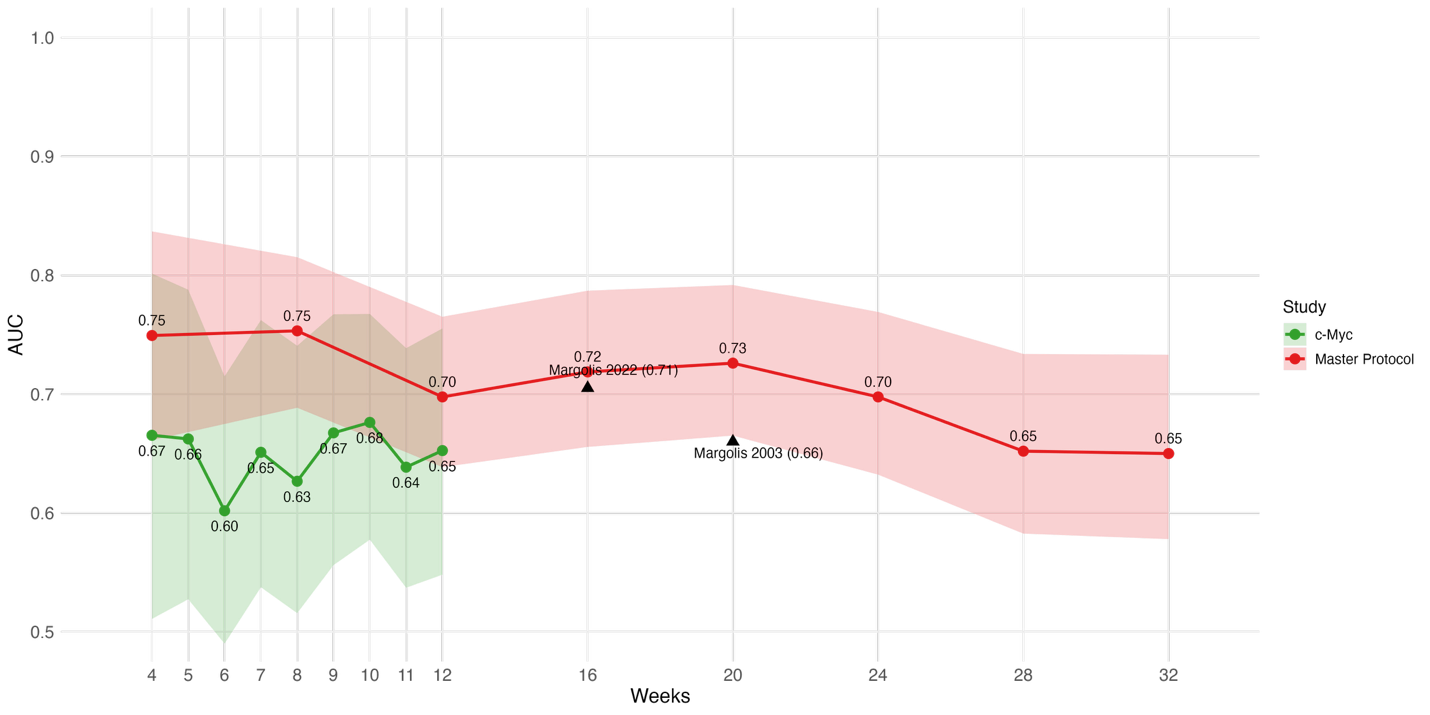


## Figure SF3a.

Beta coefficient estimates (±95% confidence intervals) from the logistic regression models for the probability of wound healing by week based on (A) wound area only (purple), (B) wound area and depth (yellow), and (C) wound area, depth, and duration (green). Top panel (solid lines) reflects models based on MP study, and bottom panel (dashed lines) reflects models based on c-Myc study.


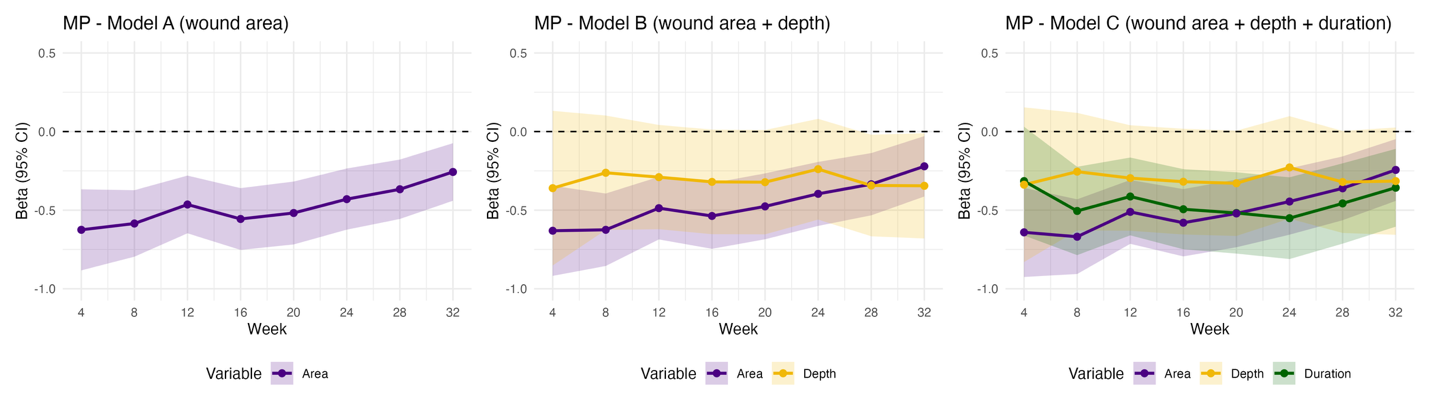


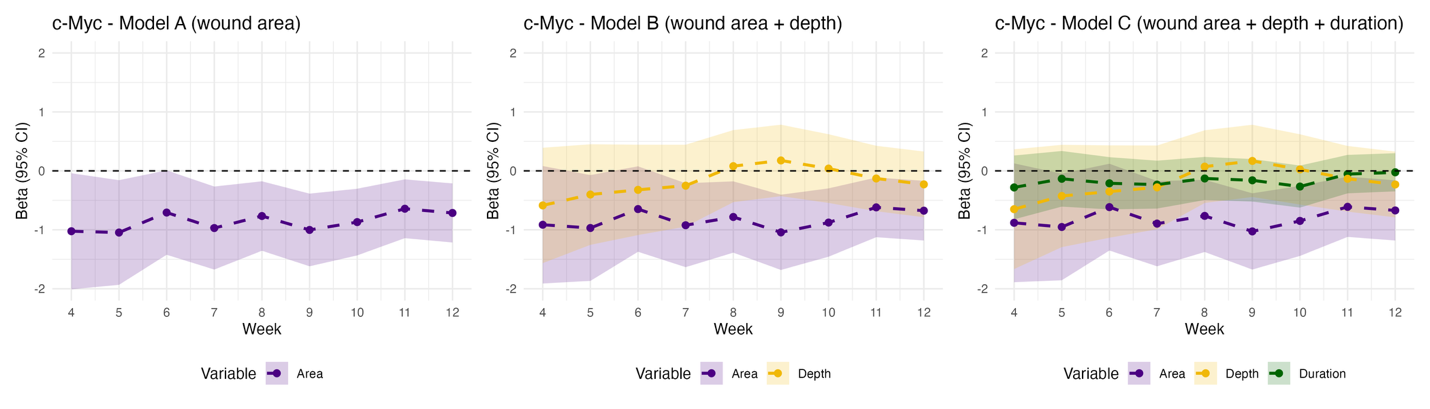


## Figure SF3b.

The predictive performance (area under the receiver operating characteristic curve, AUC, with 95% bootstrap confidence interval) of the logistic regression models for the probability of wound healing by week based on (A) wound area only (orange), (B) wound area and depth (pink), and (C) wound area, depth, and duration (purple). Results are presented separately for MP (solid line) and c-Myc (dashed line).


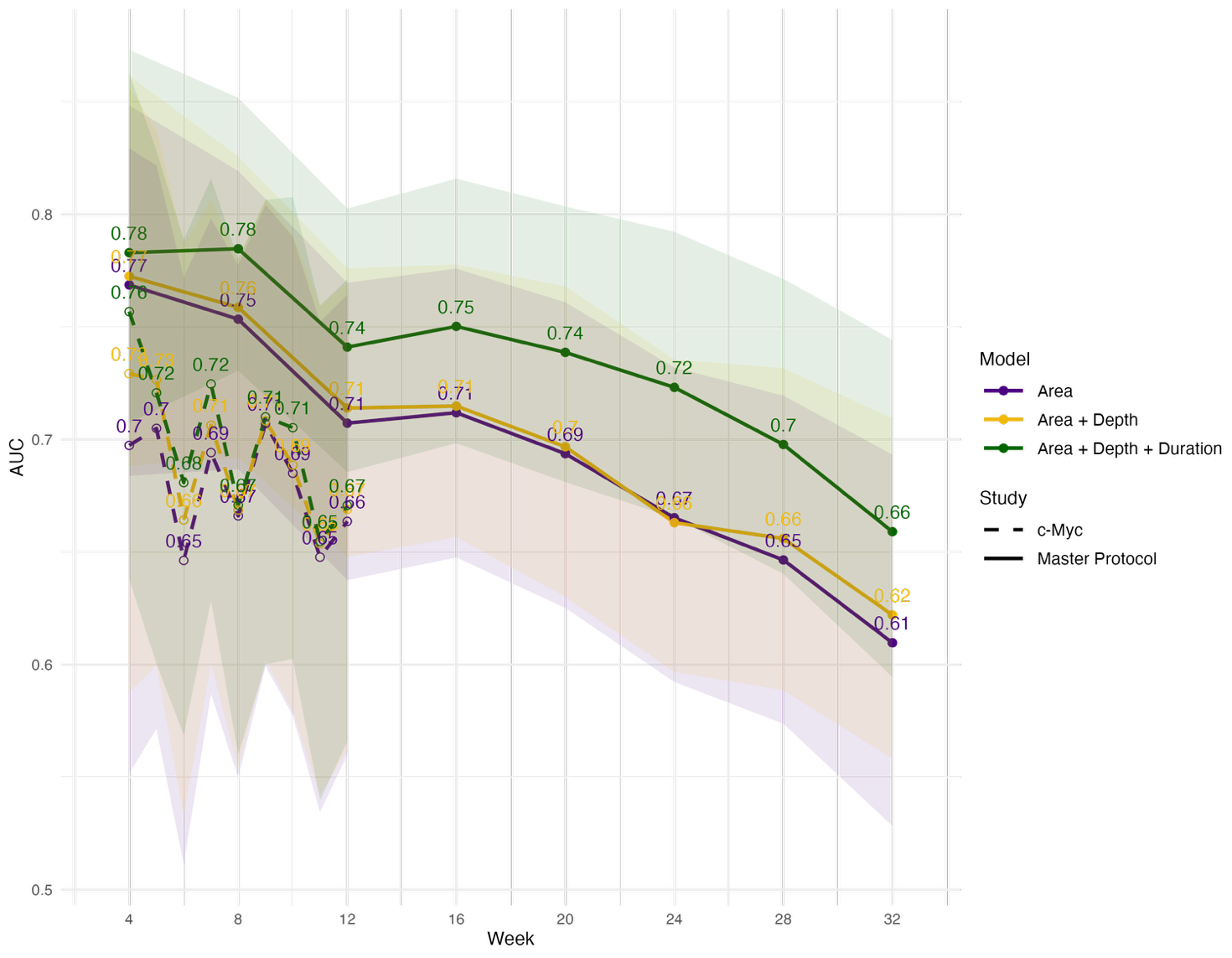


# **Supplemental Table**

## Table ST1. *Estimated healing rates from the main manuscript represented in the table*

| **Study** | **Metric** | **Week 12** | **Week 16** | **Week 20** | **Week 24** | **Week 28** |
| --- | --- | --- | --- | --- | --- | --- |
| MP | Wound Duration | -0.35  [-0.58, -0.12] | -0.44  [-0.69, -0.20] | -0.47  [-0.72, -0.22] | -0.51  [-0.76, -0.26] | -0.43  [-0.68, -0.19] |
| c-Myc | Wound Duration | -0.11  [-0.42, 0.19] | — | — | — | — |
| MP | Wound Area | -0.47  [-0.66, -0.29] | -0.58  [-0.78, -0.38] | -0.54  [-0.75, -0.34] | -0.46  [-0.66, -0.26] | -0.38  [-0.57, -0.18] |
| c-Myc | Wound Area | -0.50  [-0.95, -0.06] | — | — | — | — |
| MP | AUC | 0.72  [0.66, 0.78] | 0.74  [0.69, 0.81] | 0.74  [0.68, 0.80] | 0.72  [0.66, 0.79] | 0.69  [0.63, 0.76] |
| c-Myc | AUC | 0.64  [0.54, 0.74] | — | — | — | — |

*Note.* Output of logistic regression model (1) for the probability of wound healing over time from the main manuscript. The summary table presents beta coefficients for baseline wound duration and wound area with 95% confidence intervals, along with corresponding AUC values and 95% bootstrap confidence intervals from the model with both baseline wound duration and wound area included.

# **References**

1. Margolis DJ, Allen-Taylor L, Hoffstad O, Berlin JA. Diabetic neuropathic foot ulcers: predicting which ones will not heal. *The American Journal of Medicine*. 2003;115(8):627-631. doi:10.1016/j.amjmed.2003.06.006

2. Margolis DJ, Mitra N, Malay DS, et al. Further evidence that wound size and duration are strong prognostic markers of diabetic foot ulcer healing. *Wound Repair and Regeneration*. 2022;30(4):487-490. doi:10.1111/wrr.13019

# **Supplemental Acknowledgements**

| National Institutes of Health, National Institute of Diabetes and Digestive and Kidney Diseases | | | | |
| --- | --- | --- | --- | --- |
| Current Program Officials: | Teresa Jones, Yan Li | | | |
| Previous Program Official: | Henry Burch | | | |
|  | |  | | |
| University of Michigan Data Coordinating Center | | | | U24DK122927 |
| Current Study Members: | Cathie Spino (DCC PI), Giselle Kolenic, Peter Song, Irina Gaynanova, Wen Ye, Jordan Jahnke, Amy Kambrink, Zhongze Li, Subrahmanyam Pilli, Soumik Purkayastha, Leyao Zhang, Zheshi Zhang, Bo Yang, Rui Nie, Charlotte Xu, Junyoung Park, Shirley-Ann Saban-Quaye, Meghan Johnson, Breanna Bladowski, Gloria Morgan, Zachery DeLong, Mike Schropp, Wendy Fane, Chandrakanth Nadella, Kevin Weatherwax, Cristina Gutierrez, Meagan Marceau, Noelle Herzog | | | |
| Previous Study Members: | Brandi Gizinski, Katy Clark, Brooke Kilyanek, Tina Lucas, Vasundhara Goplani, Jennifer Mawby, Lili Zhao, Lisa Holloway, Julia Warner, Jeff Holtzman, Steve Thelen-Perry, Nazila Mirzadjahromi, Reagan Young, Miriam Michaels | | | |
| Less than 6 Months: | Linda Okoth | | | |
|  | |  | | |
| **Diabetic Foot Consortium (DFC) Clinical Research Units (CRUs; ordered by Site Number)** | | | | |
| University of California, San Francisco | | | U01DK119100 | |
| Current Principal Investigator: | | Michael S Conte | | |
| Current Research Manager / Primary Study Coordinator: | | Donna Liu, Claudio Gamboa | | |
|  | |  | | |
| University of Michigan | | | U01DK119083 | |
| Current Principal Investigators: | | Crystal Murray Holmes, Rodica Pop-Busui, Brian M Schmidt | | |
| Current Primary Study Coordinator: | | Kourtney Knoll | | |
|  | |  | | |
| University of Arizona | | | U01DK119094 | |
| Current Principal Investigators: | | Geoffrey C Gurtner, Wei Zhou | | |
| Current Primary Study Coordinator: | | Fleur Maturo | | |
|  | |  | | |
| University of Pittsburgh | | | U01DK119099 | |
| Current Principal Investigators: | | Chandan K Sen, Gayle M Gordillo, Sashwati Roy | | |
| Current Primary Study Coordinator: | | Urmila Gnyawali | | |
|  | |  | | |
| **DFC Satellite Sites (ordered by CRU Site Number)** | | | | |
| University of Southern California | | |  | |
| Current Principal Investigator: | | David Armstrong | | |
| Current Primary Study Coordinator: | | Fabiola Rodriguez | | |
|  | |  | | |
| Northwell Health | | |  | |
| Current Principal Investigator: | | Alisha Oropallo | | |
| Current Primary Study Coordinator: | | Amit Rao | | |
|  | |  | | |
| Beth Israel Deaconess Medical Center | | |  | |
| Current Principal Investigator: | | Aristidis Veves | | |
| Current Primary Study Coordinator: | | Jessica Gilman | | |
|  | |  | | |
| **DFC Inactive Sites (ordered by Site Number)** | | | | |
| University of Pittsburgh Medical Center | | | U01DK119102 | |
| Principal Investigator: | | J Peter Rubin | | |
|  | |  | | |
| University of Miami | | | U01DK119085 | |
| Principal Investigators: | | Robert Scott Kirsner, Hadar Lev-Tov, Marjana Tomic-Canic | | |
|  | |  | | |
| Indiana University | | | U01DK119099 | |
| Principal Investigators: | | Chandan K Sen, Gayle M Gordillo, Sashwati Roy | | |
|  | |  | | |
| Stanford University | | | U01DK119094 | |
| Principal Investigator: | | Geoffrey C Gurtner | | |
